# Stability Convergence in Antibody Coformulations

**DOI:** 10.1021/acs.molpharmaceut.2c00534

**Published:** 2022-10-20

**Authors:** Hongyu Zhang, Paul A. Dalby

**Affiliations:** † Department of Biochemical Engineering, UCL, WC1E 6BTLondon, U.K.; ‡ EPSRC Future Targeted Healthcare Manufacturing Hub, UCL, WC1E 6BTLondon, U.K.

**Keywords:** antibody, coformulation, protein stability

## Abstract

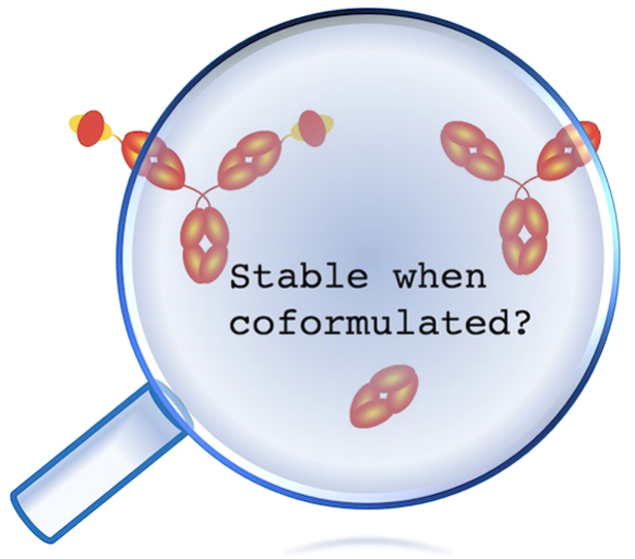

Combined administration of antibody therapeutics has proven to be beneficial for patients with cancer or infectious diseases. As a result, there is a growing trend toward multiple antibodies premixed into a single product form and delivered to patients as a fixed-dose coformulation. However, combining antibodies into a single coformulation could be challenging as proteins have the potential to interact and alter their stability and degradation profiles in the mixture, compared to that in isolation. We show that in two specific antibody–antibody coformulations, the more stable antibody component increased the stability of the less stable component, which in return destabilized the more stable component, hence exhibiting an overall convergence of stability in the coformulation.

## Introduction

Antibody-based therapies have become a major class of pharmaceutical products. The rapid growth of successful antibody therapeutic approvals is now increasingly leading toward their exploration for combined use to achieve a synergistic inhibition of therapeutic targets.^1−3^ Clinical studies of antibody combinations have shown promising improvement in a number of diseases.^4,5^ However, multiantibody therapies bring additional complexities and challenges to their innovation as products suitable for combined administration, particularly when coformulated into a single product dosage form.^6−10^

Coformulation of biologics was first achieved in medicine for blood sugar control in which short- and long-acting insulin variants or insulin and GLP-1 agonist are premixed in an injection pen before delivering to the patient.^11^ By doing so, increased patient compliance was achieved and the treatment time was reduced for hospital staff. In 2020, two antibodies against HER-2 in breast cancer were coformulated with a third protein, hyaluronidase, to create a fixed-dose subcutaneous injection form, which greatly reduced the time taken for administration compared to the traditional intravenous injection route.^12^ Moreover, a number of antibody codevelopments are in the pipeline that will potentially create coformulation products with up to 25 antibodies mixed into a single product.^13−20^ A prominent recent example is the SARS-COV-2 neutralizing antibody cocktail REGN-COV2 (Regeneron), which shows better neutralization than single-antibody treatments.^21^ Antibody cocktail products seem to be of greater advantage in the mitigation of viral infection as binding to multiple antigenic sites on the viral surface spike protein reduces the chances of epitope escape.^22,23^

The creation of antibody coformulations can be more challenging than the combination of small molecules. Stability of antibodies is susceptible to changes in temperature, mechanical force, pH, ionic strength, and protein concentration.^24−27^ Mixtures of antibodies and other therapeutic or excipient proteins could also create the risk of heterogeneous aggregation—an irreversible change of protein structure leading to immunogenic species and reduction of biological activity.^6,7,28,29^ Therefore, the impact of coformulation on the individual stability of the antibodies must be carefully investigated during the development of coformulated products.

**Figure 1:**
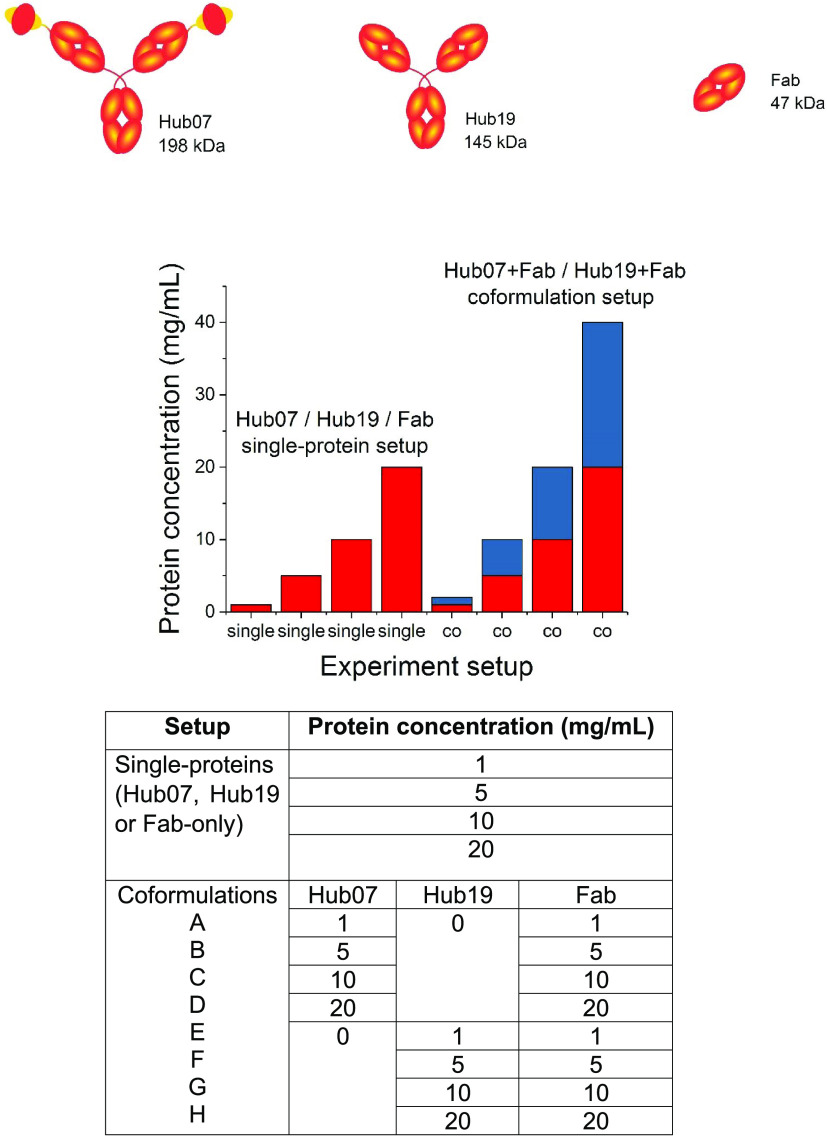
Setup for Hub07, Hub19, and Fab in single-protein and coformulation experiments. Hub07 is a bispecific antibody with two distinct scFV in fusion with an IgG1 framework. Hub19 is a typical full IgG1 antibody. Fab is a therapeutic fragment isolated from a full IgG1. Hub07, Hub19, and Fab were each characterized at 1, 5, 10, and 20 mg/mL in a single-protein setup as reference systems. Hub07 and Hub19 at 1–20 mg/mL were, respectively, mixed with the Fab at a 1:1 mass ratio for the coformulation measurements. Each row shown in coformulations A–H is a separate coformulation.

Previously, we have shown that a specific therapeutic antigen-binding fragment (Fab) could stabilize an intact IgG1 antibody that had the same CDR sequences, potentially by abrogating adverse self-interactions between the Fc regions of IgG1.^30^ It is therefore of interest to determine whether the same Fab could also stabilize other antibodies or even bispecific designs, regardless of the target antigen. Here, we examined the degradation profiles of an IgG1 antibody-scFv bispecific fusion protein (denoted “Hub07”) and a full IgG1 antibody (denoted “Hub19”) (Figure 1) and the impact of their coformulation with the Fab. The CDR regions of these antibodies were all different as they were developed to bind to different antigens, which models the most likely antibody coformulation scenario where each one targets different antigens, or different epitopes of the same antigen. The antibodies to be mixed were first characterized individually from 1 to 20 mg/mL as reference systems. Next, Fab was mixed 1:1 with Hub07 or Hub19 at the same individual concentrations, and the stabilities of antibodies in these coformulations were characterized from their monomer-loss kinetics (Figure 1). Again, we found that the Fab stabilized Hub07 and Hub19 from the monomer loss. However, in contrast to the previous study, Hub07 and Hub19 molecules destabilized the Fab slightly such that the overall stability of the mixtures converged to a point between the stabilities of the individual mixing components.

## Experimental Section

### Materials

The *Escherichia coli* strain W3110 containing pTTOD A33 IGS2 for the Fab expression was obtained from UCB (Slough, UK). Antibodies Hub07 and Hub19 are provided by AstraZenecca (Cambridge, UK). Size-exclusion chromatography (SEC) column was purchased from Agilent (Stockport, UK). PBS buffer is purchased from Sigma-Aldrich (Poole, UK).

### Methods

#### Protein Production and Purification

The Fab was produced from a pilot-scale expression in a 30 L fermenter (BIOSTAT Cplus, Sartorius, Goettingen, Germany) and purified using AKTA-based liquid chromatography as described elsewhere.^26^ The purified protein was dialyzed in PBS buffer at 4 °C overnight using Dialysis Cassettes, 10K MWCO (Thermo Scientific, 66 811) and concentrated up to 40 mg/mL using ultracentrifugation prior to dilution into desired concentrations.

Hub07 and Hub19 were loaded onto a gel filtration column (HiLoad Superdex 75, GE Healthcare) equilibrated with PBS buffer to remove minor large molecular weight species. The purified protein was concentrated up to 40 mg/mL using ultracentrifugation and sterile-filtered (0.2 μm) prior to dilution into desired concentrations with sterile-filtered buffers. Protein concentrations were determined from A280 nm measurements on a NanoDrop One system (ThermoFisher Scientific). The extinction coefficient for each protein was provided by UCB for the Fab (1.40 mL/mg/cm) and AstraZeneca for Hub07 and Hub19 (1.49 and 1.70 mL/mg/cm), determined from their respective Trp, Tyr, and Cys contents in their protein sequences.

#### Sample Preparation

For the measurements of coformulated proteins, Fab was mixed with Hub07 or Hub19 at a mass ratio of 1:1. We chose to use a constant mass rather than a constant molar ratio to avoid large changes in the partial specific volume occupied by the proteins, that would otherwise introduce changes in stability through macromolecular crowding effects. The concentrations of each protein in the mixture were 1, 5, 10, and 20 mg/mL (5.1, 25.4, 50.8, and 101.5 μM) for Hub07, 6.9, 34.5, 69.1, and 138.1 μM for Hub19, and 21.1, 105.5, 211.1, and 422.1 μM for the Fab, as calculated from their respective molecular weights. Thus, the stoichiometries are approximately 4.2:1 and 3.1:1 for Fab:Hub07 and Fab:Hub19 coformulations, respectively. Fab, Hub07, and Hub19 were also measured individually at 1, 5, 10, and 20 mg/mL as references. All measurements were carried out in PBS buffer at pH 7.4.

#### Thermal Stability Measurement

The thermal stabilities of Fab, Hub07, and Hub19 in their single or coformulation forms were each measured using a UNit system (UNCHAINED LABS, Pleasanton). Each sample well in the cartridge was loaded with 9 μL of the protein of respective concentration. The protein was step-heated from 20 to 95 °C at 1 °C/min and with 30 s equilibration at each temperature. The fluorescence signal was recorded as the BCM (Barycentric Mean) of each spectrum, which was calculated by the instrument software and plotted against temperature. Each experiment was measured in triplicate and averaged.

The data were fitted using a two-state unfolding model (eq 1) to extract the apparent midpoint of unfolding transitions (*T*_m,app_)
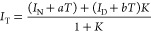
1with an equilibrium constant for the transition between the native and denatured state
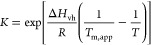
where *T* is the experimental temperature; *T*_m,app_ is the temperature at which the protein is half-denatured; *I*_T_, *I*_N_, and *I*_D_ are the spectroscopic signals of the protein at each given temperature, at the native and at the fully denatured state, respectively. *a* and *b* are the baseline slopes of the native and denatured regions of the curve. Δ*H*_vh_ is the van’t Hoff enthalpy and *R* is the gas constant. All temperature terms in this equation are absolute temperatures in Kelvin.

#### Degradation Kinetics Measurement Using SEC-HPLC

For single-protein measurements, Hub07, Hub19, and Fab at each concentration, and also the coformulated samples, were incubated at 4, 37 and 50 °C in tight-lid Eppendorf tubes to minimize evaporation. The results of 4 °C samples were used as a control reference for 37 and 50 °C stressed samples. The samples containing Hub19 were stressed for 40 days and the samples containing Hub07 were stressed for 60 days.

Monomer retention of single and coformulation samples were measured using size-exclusion chromatography (SEC) to assess the level of protein degradation in each formulation. Aliquots of 60 μL were taken at each time point from the pool and centrifuged at 11 000*g* for 45 min at 4 °C to remove large insoluble aggregates. The supernatant (50 μL) was transferred into glass vials, held at 4 °C, prior to analysis on a Zorbax-GF250 column. For each measurement, 10 μL of the sample was injected to the column at 1 mL/min on an HPLC instrument (1200 series, Agilent, UK) using 200 mM sodium phosphate pH 7.0 as the mobile phase with the column at room temperature. Due to the limit of the SEC method, it is possible that a fraction of reversible soluble aggregates was dissociated back into the monomer upon dilution into the column. Therefore, the actual monomer retention was potentially less than the reported values. Elution profiles at 280 nm were averaged over at least three repeated measurements. Peaks were fitted to a modified Gauss equation (eq 2) in OriginPro 2016 (OriginLab, UK) to obtain the peak area
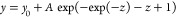
2where 

In these equations, *y* is the absorbance, *y*_0_ is the offset of the chromatogram, *x* is the elution time, *xc* is the center of the peak, *w* is the width, and *A* is the amplitude. Then, the area under the peak was obtained by integration.

The change in the relative concentration of the monomer and other degradation species was calculated by subtracting the monomer peak area measured for the 50 °C stressed samples from those of the 4 °C samples and normalized over the peak area at day 0
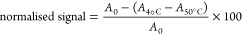
3where *A*_0_ is the peak area at time zero; *A*_4°C_ and *A*_50°C_ are the peak areas of 4 and 50 °C at each given incubation time.

## Results

### Monomer Loss of Hub07, Hub19, and Fab in Single-Protein Form

**Figure 2:**
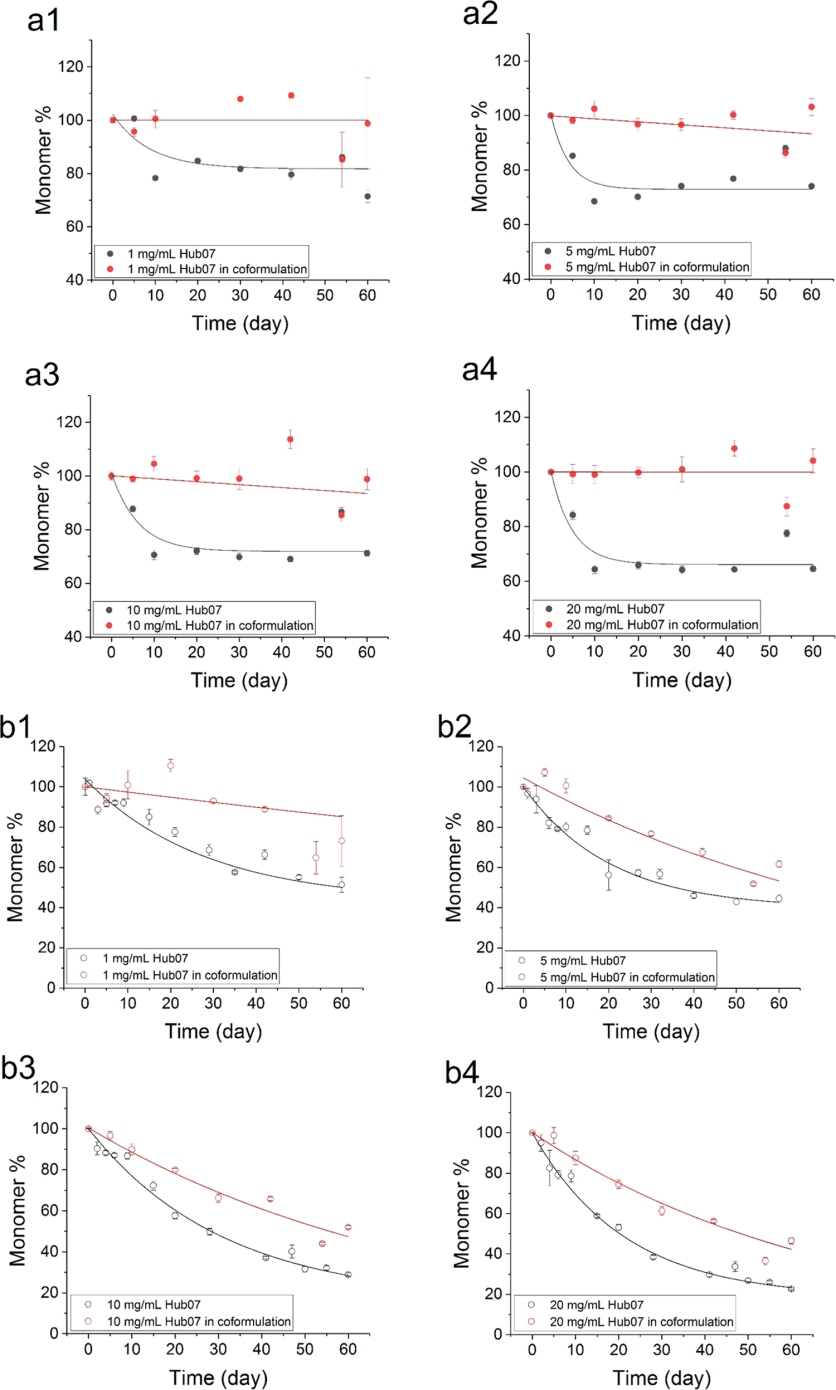
Monomer retention of Hub07 in single-protein and coformulation setup in PBS buffer (pH 7.4). Changes in the monomer stressed at 37 °C are shown as filled circles (a1–a4) and those stressed at 50 °C are shown as open circles (b1–b4). Data are either fitted to a single-exponential decay equation (all 50 °C measurements and single-protein data at 37 °C) or to a linear equation (coformulation data at 37 °C). Error bars shown are standard deviations from triplicate measurements at each time point.

**Figure 3:**
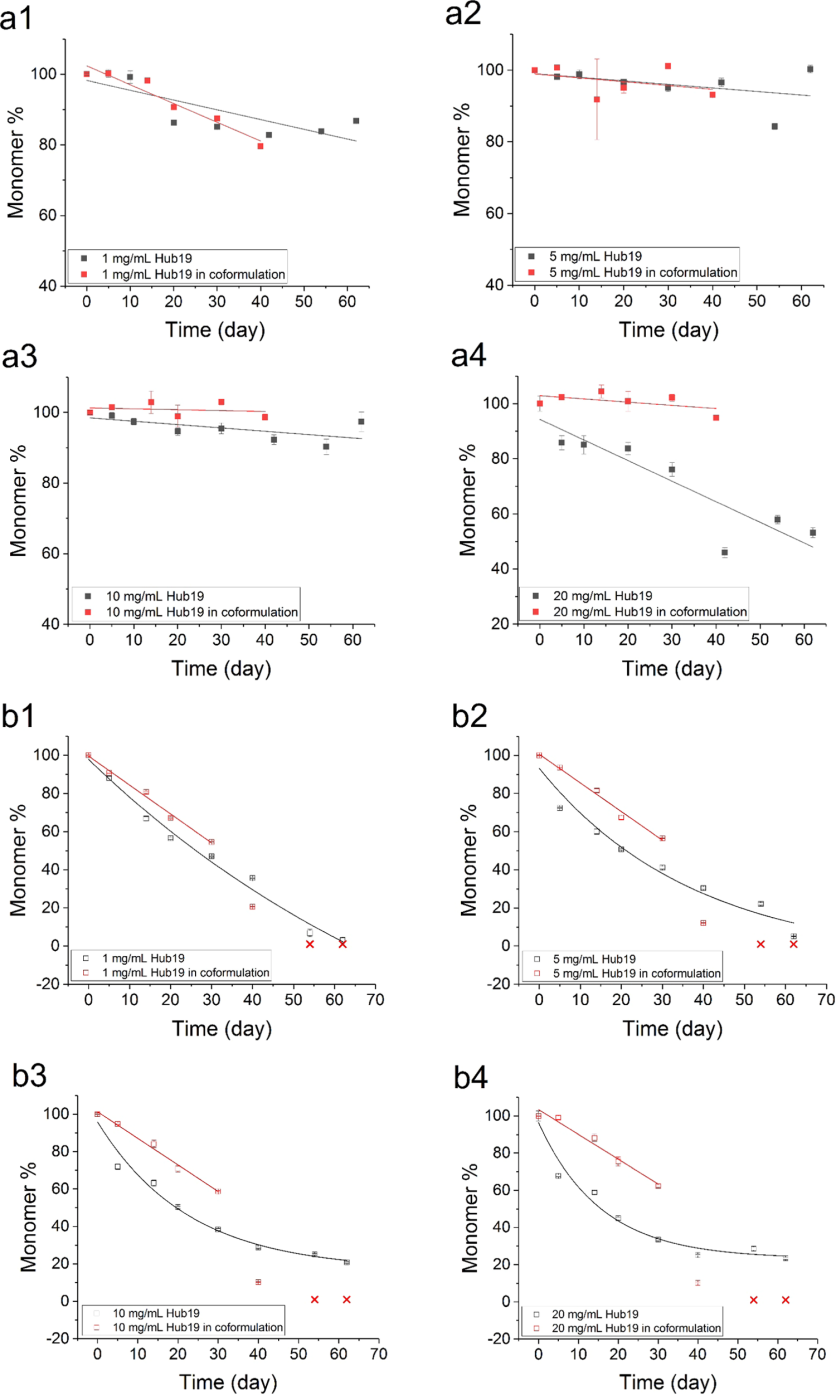
Monomer retention of Hub19 in single-protein and coformulation setup in PBS buffer (pH 7.4). Changes in the monomer stressed at 37 °C are shown as filled squares (a1–a4) and those stressed at 50 °C are shown as open squares (b1–b4). Data are either fitted to a single-exponential decay equation (single-protein data at 50 °C) or to a linear equation (single protein at 37 °C and coformulation data at both 37 and 50 °C). A nominal 1% monomer is shown as red crosses at days 50 and 60 for coformulation measurements at 50 °C, where monomer measurements could no longer be obtained due to excessive aggregate formation. Error bars shown are standard deviations from triplicate measurements at each time point.

**Figure 4:**
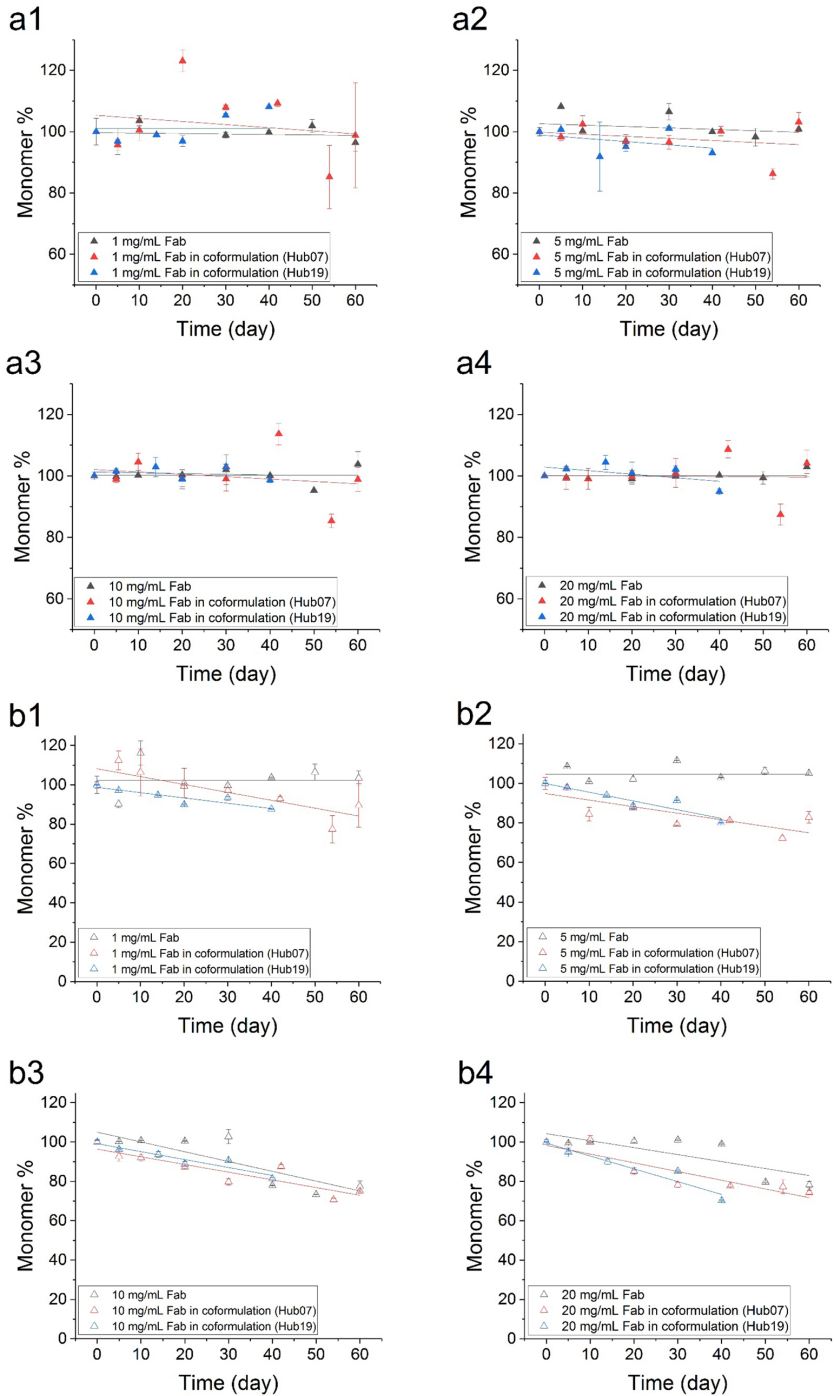
Monomer retention of the Fab in single-protein and coformulation setup in PBS buffer (pH 7.4). Changes in the monomer stressed at 37 °C are shown as filled triangles (a1–a4) whereas those stressed at 50 °C are shown as open triangles (b1–b4). Data were fitted to a linear equation. Error bars shown are standard deviations from triplicate measurements at each time point.

**Figure 5:**
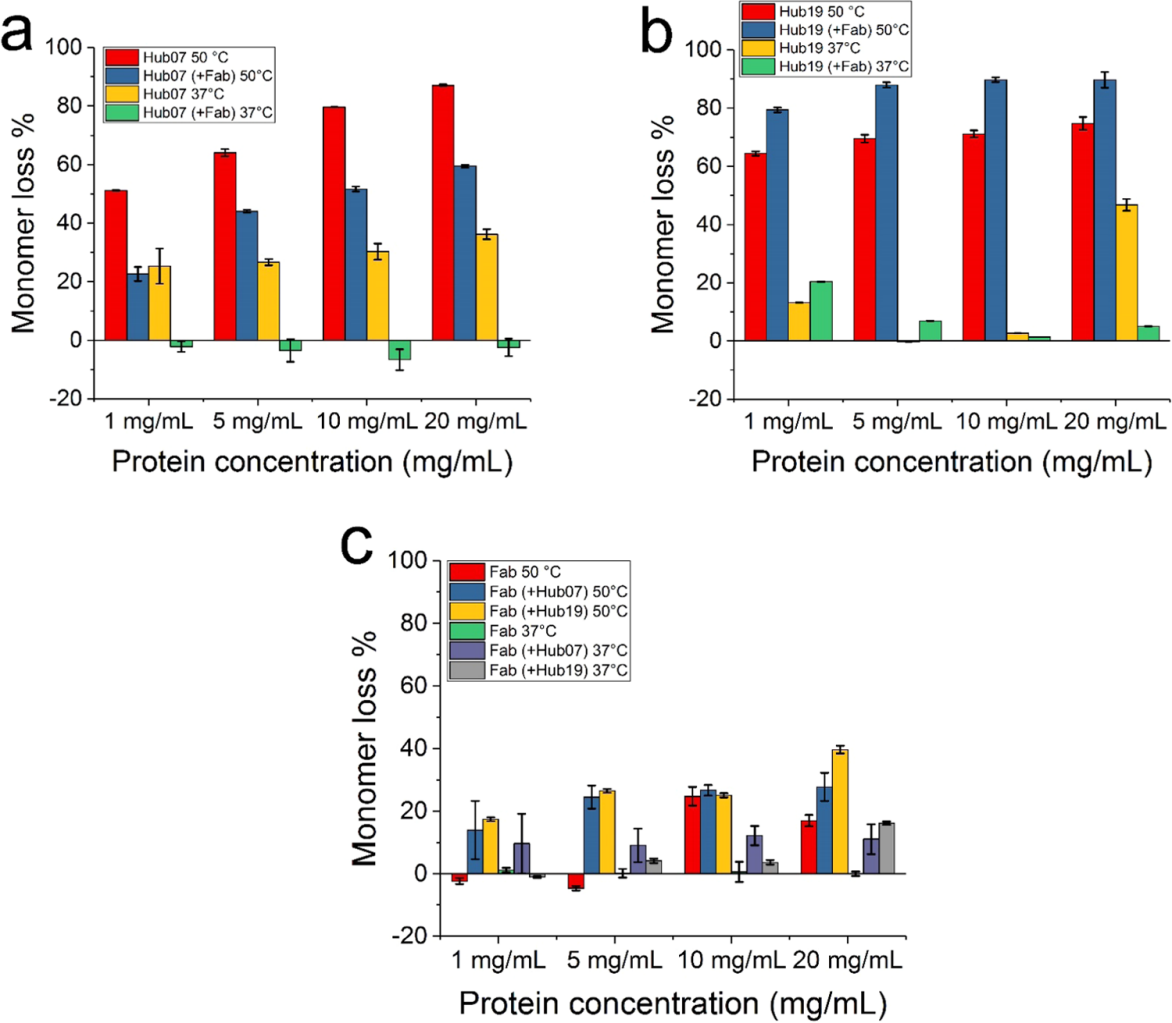
Monomer loss of Hub07, Hub19, and Fab in single-protein and coformulation measurements in PBS buffer (pH 7.4). Error bars are standard errors from three repeats.

Hub07, Hub19, and Fab were each formulated at 1, 5, 10, and 20 mg/mL and analyzed for the loss of the monomer using standard SEC while being stressed at 37 or 50 °C in PBS buffer (pH 7.4) for up to 60 days (Figure 2, 3, 4). Some curves did not reach plateaus at this time point, but we decided not to make further measurements to avoid artifacts (evaporation, etc.) affecting the results. The aggregate was the primary degradation product though minor levels of fragments were also present at the end of the stress experiment (Figure S1). Hub07 showed a typical exponential decay of the monomer species at 37 and 50 °C. The kinetic parameters were extracted by fitting the data to a single-exponential equation (Table 1). The total monomer loss of Hub07 after 60 days increased from 25 to 35% at 37 °C and from 50 to 90% at 50 °C as the protein concentration increased from 1 to 20 mg/mL (Figure 5 and Table 2).

**Table 1:** Kinetics for the Monomer Loss of Hub07, Hub19, and Fab in Single-Protein and Coformulation Measurements^a^

		Hub07			Hub07 (+Fab)		
stress temperature	concentration	*A*	*k* (day^–1^)	*v*_initial_(% day^–1^)	*v*(% day^–1^)		
37 °C	1 mg/mL	27 ± 5	0.06 ± 0.02	1.6 ± 0.7	0.01 ± 0.01		
	5 mg/mL	28 ± 2	0.20 ± 0.03	5.5 ± 0.9	0.06 ± 0.04		
	10 mg/mL	31 ± 4	0.16 ± 0.03	5.1 ± 1.2	0.08 ± 0.03		
	20 mg/mL	37 ± 2	0.17 ± 0.02	6.5 ± 0.7	0.03 ± 0.03		
	concentration	*A*	*k* (day^–1^)	*v*_initial_(% day^–1^)	*A*	*k* (day^–1^)	*v*_initial_(% day^–1^)
50 °C	1 mg/mL	99 ± 1.5	0.012 ± 0.003	1.2 ± 0.1	105 ± 3	0.005 ± 0.0004	0.5 ± 0.04
	5 mg/mL	94 ± 2	0.016 ± 0.001	1.5 ± 0.1	107 ± 1	0.011 ± 0.0005	1.2 ± 0.05
	10 mg/mL	95 ± 0.5	0.026 ± 0.001	2.5 ± 0.08	101 ± 2	0.012 ± 0.0002	1.3 ± 0.03
	20 mg/mL	93 ± 2	0.033 ± 0.0015	3.1 ± 0.15	102 ± 2	0.015 ± 0.0005	1.6 ± 0.06

		Hub19			Hub19 (+Fab)
stress temperature	concentration	*v*(% day^–1^)			*v*(% day^–1^)
37 °C	1 mg/mL	0.28 ± 0.01			0.50 ± 0.01
	5 mg/mL	0.1 ± 0.01			0.14 ± 0.01
	10 mg/mL	0.09 ± 0.01			0.03 ± 0.01
	20 mg/mL	0.75 ± 0.04			0.12 ± 0.01
		*A*	*k* (day^–1^)	*v*_initial_(% day^–1^)	*v*(% day^–1^)
50 °C	1 mg/mL	101 ± 1	0.032 ± 0.001	3.3 ± 0.1	1.52 ± 0.01
	5 mg/mL	92 ± 0.3	0.031 ± 0.001	2.8 ± 0.1	1.50 ± 0.01
	10 mg/mL	90 ± 0.4	0.027 ± 0.0002	2.5 ± 0.02	1.42 ± 0.01
	20 mg/mL	87 ± 0.3	0.028 ± 0.0003	2.5 ± 0.03	1.33 ± 0.01

		Fab	Fab (+Hub07)	Fab (+Hub19)
stress temperature	concentration	*v*(% day^–1^)	*v*(% day^–1^)	*v*(% day^–1^)
37 °C	1 mg/mL	0.01 ± 0.01	0.4 ± 0.1	0.01 ± 0.01
	5 mg/mL	0.05 ± 0.01	0.1 ± 0.1	0.06 ± 0.01
	10 mg/mL	0.03 ± 0.02	0.16 ± 0.03	0.05 ± 0.01
	20 mg/mL	0.002 ± 0.0015	0.22 ± 0.03	0.28 ± 0.01
50 °C	1 mg/mL	0.001 ± 0.001	0.4 ± 0.1	0.27 ± 0.01
	5 mg/mL	0.01 ± 0.01	0.3 ± 0.1	0.44 ± 0.01
	10 mg/mL	0.5 ± 0.07	0.38 ± 0.01	0.4 ± 0.01
	20 mg/mL	0.35 ± 0.04	0.4 ± 0.1	0.65 ± 0.02

a The rate constant (k, day^–1^) obtained from the exponential model and the rate of monomer decay (*v*,% day^–1^) obtained from the linear model are shown. The initial rate was obtained by *v*_initial_ = *A* * *k*. Errors quoted are standard deviation of the fit from three repeats.

**Table 2:** Monomer Loss of Hub07, Hub19, and Fab in Single-Protein and Coformulation Setup^a^

single protein				
stress temperature	concentration	Hub07	Hub19	Fab
37 °C	1 mg/mL	25 ± 6%	13 ± 0.1%	1 ± 1%
	5 mg/mL	27 ± 1%	0 ± 0.01%	0 ± 1%
	10 mg/mL	30 ± 3%	3 ± 0.1%	1 ± 3%
	20 mg/mL	36 ± 2%	47 ± 2%	0 ± 1%
50 °C	1 mg/mL	51 ± 0.1%	64 ± 1%	–2 ± 1%
	5 mg/mL	64 ± 1%	70 ± 1%	–5 ± 1%
	10 mg/mL	80 ± 0.1%	71 ± 1%	25 ± 3%
	20 mg/mL	87 ± 0.3%	75 ± 2%	17 ± 2%

coformulation					
stress temperature	concentration	Hub07 (+Fab)	Hub19 (+Fab)^b^	Fab (+Hub07)	Fab (+Hub19)
37 °C	1 mg/mL	–2 ± 2%	20 ± 0.1%	10 ± 10%	–1 ± 1%
	5 mg/mL	–4 ± 4%	7 ± 0.02%	9 ± 5%	4 ± 1%
	10 mg/mL	–7 ± 4%	1 ± 0.01%	12 ± 3%	4 ± 1%
	20 mg/mL	–3 ± 3%	5 ± 0.1%	11 ± 5%	16 ± 1%
50 °C	1 mg/mL	23 ± 2%	79 ± 1%	14 ± 9%	18 ± 1%
	5 mg/mL	44 ± 1%	88 ± 1%	25 ± 4%	27 ± 1%
	10 mg/mL	52 ± 1%	90 ± 1%	27 ± 2%	25 ± 1%
	20 mg/mL	60 ± 1%	90 ± 3%	28 ± 5%	40 ± 1%

a The data were calculated on day 60 from their respective linear or exponential kinetic model.

b Monomer loss of Hub19 at 50 °C in single and coformulation is calculated and presented as the SEC data measured on day 40.

The monomer loss for Hub19 was linear at 37 °C where a maximum of 13–47% degradation was observed in 60 days. This became more clearly exponential at 50 °C as the maximum extent of degradation reached 65–75% (Figure 3 and Table 2). The extracted initial rates of monomer loss at 37 °C and the rate constants at 50 °C are shown in Table 2. At 37 °C, Hub19 lost up to 13% monomer at 1 mg/mL, and no more than 5% at 5 and 10 mg/mL. At 20 mg/mL, Hub19 showed up to 47% monomer loss at the end of day 60. The stability of Hub19 was considerably reduced at 50 °C with 75% monomer loss at day 60.

Fab, in contrast, had greater stability than Hub07 and Hub19 as it showed very little monomer loss at both 37 and 50 °C over 60 days, while very mild loss of the monomer (17–25%) was only observed for the combination of 50 °C and higher concentrations (10 and 20 mg/mL) (Figures 4, 5 and Table 2). The kinetic data for the Fab were fitted to a linear equation to extract the initial rates of monomer loss (Table 1).

### Monomer Loss of Hub07 and Hub19 in Coformulations with Fab

In coformulations, Hub07 and Hub19 showed a decreased tendency to the monomer loss compared to single-protein setups. At 37 °C, the monomer loss of Hub07 slowed to 0% or a barely discernible change over 60 days (Figure 2a1–a4 and Table 2) compared to the 25–35% monomer loss in the single-protein experiment. At 50 °C, no change of the kinetic profile was seen for the degradation of Hub07 in the coformulation compared to the single-protein form. Thus, the coformulation data were fitted to the same single-exponential equation as for the single-protein data, showing up to 50% reduction in the rate constant and initial rate for the degradation of Hub07 in the coformulations (Table 1). The monomer loss after 60 days at 50 °C for Hub07 was reduced from 50 to 90% in the single-protein system to 20–60% in the coformulations. In each case, the rates and final monomer losses both increased as the protein concentration increased from 1 to 20 mg/mL (Table 2).

The monomer-loss kinetics for the coformulated Hub19 at 37 °C continued to fit well to a linear decay (Figure 3a1–a4 and Table 1). Higher Hub19 concentrations were stabilized the most by the presence of the Fab, showing a greater difference in the rate of the monomer loss relative to that in the single-protein form, as the Hub19 concentration increased from 1 to 20 mg/mL. Approximately 20% of the Hub19 monomer was lost in the 1 mg/mL coformulation at 37 °C after day 40. This reduced to only a 5% loss of Hub19 in the 20 mg/mL coformulation, again showing a concentration-dependent increase in stability in the presence of an equal mass concentration of the Fab.

Under stress at 50 °C, the initial rate and extent of the Hub19 monomer loss was again reduced by the presence of the Fab up to day 30 and by a greater extent at higher protein concentrations. However, this now appeared to introduce a lag phase prior to a sharp drop in the monomer loss at between days 30 and 40, giving rise to two separate kinetic regimes. From day 50 at 50 °C, it was difficult to measure the Hub19 content in the coformulations due to severe aggregation. This was shown by an opalescent appearance and significantly increased viscosity of the sample such that centrifugation and pipette sampling were not possible prior to SEC analysis. Given that the monomer loss on day 40 had reached 80% at 1 mg/mL and 90% at 20 mg/mL, it is likely that this was at or close to 100% at day 50 (hypothetically marked as x symbols with a nominal 1% monomer in Figure 3b1–b4). As a result, only the data from 0 to 30 days were fitted to linear decay equations, representing the initial lag-phase decay kinetics.

The conformational and colloidal stabilities of Hub07 and Hub19 in coformulations were also probed using intrinsic fluorescence and static light scattering in a thermal unfolding experiment (Figures S2 and S3). The overall conformational stability was measured by fitting to the dominant transition. Given that the antibody denaturation is irreversible, we termed this an apparent thermal midpoint (*T*_m,app_). Hub07–Fab and Hub19–Fab mixtures showed an increase in *T*_m,app_ compared to the single-protein measurements including that of the Fab. The experimental values of *T*_m,app_ in Hub07–Fab and Hub19–Fab were closer to that of the Fab compared to the modeled values from fitting the mathematical average of Hub07, Hub19, and Fab single-protein data. This indicated that these coformulation systems did not cause catastrophic stability loss due to an increase in the overall protein concentration and composition, but on the contrary became stabilized in the presence of the Fab. However, there remained weak signals from the earliest transitions of Hub07 and Hub19 at approximately 60–65 °C that were not possible to fit.

The static light-scattering measurements monitored by 266 and 473 nm intensity showed the formation of small and large aggregates, respectively (Figure S3). The decrease of 266 nm scattering intensity was often followed by an increase in the 473 nm scattering intensity. We propose that this shows a transition from small to large aggregates as the proteins were heat-denatured. In many cases, the 473 nm intensity dropped at high temperatures because presumably the protein was precipitated out of the solution and so out of the light path. In general, the increase in protein concentration did not change the overall onset temperature of aggregation (*T*_agg_) for Hub07 alone, Hub19 alone, Fab alone, or for the coformulated Fab and Hub19, though it did lead to the formation of larger aggregates. The Fab was ∼10 °C more stable than Hub07 and Hub19.

For Hub07 and Fab coformulation, the *T*_agg_ decreased from approximately 72–60 °C, as the protein concentration was increased. This indicated an overall stability increase in the coformulation at 1 mg/mL, compared to Hub07 alone which had a *T*_agg_ of ∼60 °C and brought it closer to that of the Fab (∼70 °C). However, Hub07 in the coformulation was less stabilized at 20 mg/mL, indicating an interaction between Hub07 and Fab that stabilized Hub07 at 1 mg/mL but less so at 20 mg/mL. This is consistent with only a slight improvement in monomer-loss kinetics at 50 °C compared to a much larger difference at 37 °C, which is much further below the *T*_agg_ values.

Coformulated Hub19 and Fab at 1 and 5 mg/mL gave rise to two scattering transitions at temperatures corresponding to those observed for Hub19 and Fab alone, at 60 and 70 °C. This suggests that Hub19 and Fab aggregated largely independently at these concentrations. The coformulated Hub19 was slightly stabilized with a *T*_agg_ above 60 °C compared to ∼58 °C in the isolated form, consistent with the stabilizing effect of the Fab on Hub19 observed in the monomer-loss kinetics. At 10 and 20 mg/mL, the independence of Hub19 and Fab aggregation in the coformulation is less clear, but again the coformulated Hub19 *T*_agg_ remained slightly above 60 °C.

### Greater Monomer Loss of the Fab in the Coformulation with Hub07 or Hub19

Despite the increase in stabilities of Hub07 and Hub19 due to 1:1 addition of the Fab, the coformulations in return led to some slight reductions in the Fab stability. Fab had essentially 0% monomer loss over 60 days for 1–20 mg/mL at 37 °C, and the coformulations with Hub07 and Hub19 had some minor impact on this (Figure 5 and Table 2). At 50 °C, a clear impact from the coformulations on Fab stability became apparent. Fab lost 15–40% of its monomer after 40–60 days in the coformulations with Hub07 and Hub19 at all concentrations, compared to 0% loss at 1–5 mg/mL and 25% at 10–20 mg/mL for the Fab alone. Interestingly, the monomer loss for the Fab on its own at 10–20 mg/mL also occurred only after a lag period of 30–40 days, compared to a continuous decrease in the Fab coformulated with Hub07 or Hub19.The average rate of change in the monomer species of the Fab over the experimental period was obtained by fitting to a linear equation (Figure 4) to extract the rates shown in Table 1.

### Reaction Order Suggests Multiple Aggregation Pathways

The initial rates of degradation (Table 1) of Hub07, Hub19, and Fab in single-protein and coformulation setup were plotted against protein concentration (Figure S4) to determine the reaction order (RO) of each degradation reaction from their slopes. Integer RO values suggest the number of protein molecules involved in the rate-determining step of the aggregation process, for example, a RO of 1 would indicate a unimolecular reaction, such as protein unfolding or a coformational change, as the rate-limiting step toward aggregation. A RO of 2 would indicate a bimolecular reaction as rate-limiting. A fractional reaction order of 0 < RO < 1 would indicate that the reaction rate is partially limited by an independent factor such as an available surface area whereby the monomer loss proceeds through an interaction with that surface. Other fractional reaction orders of RO > 1 indicate more complex combinations of the above and/or a number of parallel pathways.

**Figure 6:**
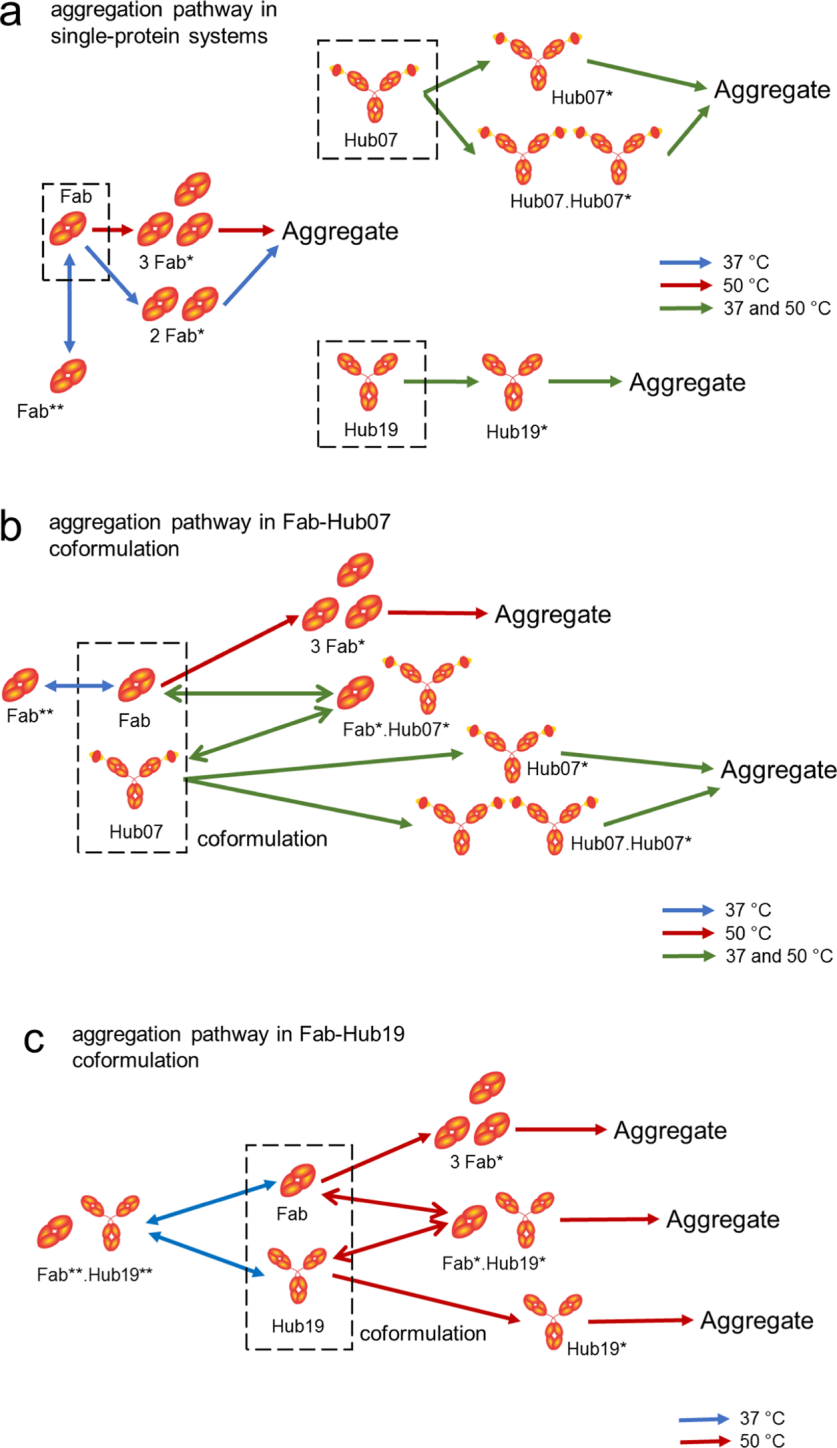
Proposed aggregation mechanism of Hub07, Hub19, and Fab in (a) single-protein, (b) Fab–Hub07 and (c) Fab–Hub19 coformulations. Solid arrows represent the degradation pathways in single-protein systems whereas dashed arrows indicate additional pathways in coformulation systems. Blue arrows indicate the reaction pathway at 37 °C and red arrows indicate the pathway at 50 °C. Green arrows indicate common pathways for both temperatures. Fab* is a partially unfolded species of the Fab that was formed prior to aggregation. Hub07 and Hub19 are also proposed to form partially unfolded species (Hub07* and Hub19*) before aggregation. The formation of Fab*, Hub07*, and Hub19* from their native states is proposed to be the rate-limiting step of degradation. At 37 °C, an off-pathway species Fab** was formed from native-state Fab, which interacts with Hub19 in the coformulation to form a Fab**.Hub** off-pathway species.

At 37 °C, the ROs of Hub07 and Hub19 were 1.3 ± 0.14 and 1.2 ± 0.35 for their single-protein measurements (Figure S4). The rate-limiting step for the monomer loss of Hub07 was essentially unimolecular, such as would be expected from an unfolding event, but with a small dependence on a parallel higher-order reaction such as a bimolecular pathway. A hypothetical example of this is shown in Figure 6B. However, Hub19 actually appeared to have a fractional rate order at low concentrations, with a sharp rate increase at 20 mg/mL.

The RO for the Fab-only formulation at 37 °C was 1.5 ± 0.5 for the lower concentration range, but a mechanism switch occurred at the highest concentration, which suppressed the rate of the monomer loss and led to an apparently lower reaction order. The RO of 1.5 ± 0.5 indicated a monomolecular reaction with some possibility of a contribution from a parallel bimolecular reaction as the rate-limiting step for the Fab monomer loss (Figure 6).

The ROs for Hub07 and Hub19 at 50 °C remained similar to those at 37 °C at 1.4 ± 0.1 and 0.93 ± 0.03, respectively, suggesting that the increased temperature did not fundamentally change the mechanism for the monomer loss. By contrast, the RO for the Fab increased to 2.7 ± 0.8 at 50 °C, indicating a shift toward higher-order species formation in the rate-limiting step for the monomer loss. For comparison, the RO for the Fab at 65 °C was previously found to be 0.7, but under those conditions ∼5% of the protein had already globally unfolded, which would therefore promote the lower-order reaction through unimolecular unfolding.^31^

The reaction order for Hub07 was unaffected by the coformulations despite the overall decrease in rates in the presence of the Fab. However, Hub07 affected the RO of the Fab, which decreased from 1.5 ± 0.5 to 0.89 ± 0.1 at 37 °C and from 2.7 ± 0.8 to 1.1 ± 0.1 at 50 °C, along with increases in the Fab monomer-loss rate at 37 and 50 °C. Thus, the presence of Hub07 appeared to promote the unimolecular pathway of Fab unfolding, while also inhibiting the higher-order reaction. As the Hub07 monomer loss was reduced by the presence of the Fab in all conditions, it appears that their interaction in solution (hypothetical species Fab*–Hub07* in Figure 6) slowed the rate of unfolding for Hub07 and blocked Fab–Fab interactions. In addition, this slightly increased the Fab unfolding rate.

For Hub19, the RO was unaffected by the presence of the Fab at 50 °C, while the rate of Hub19 loss was slightly slowed. At 37 °C and low concentrations, the RO for Hub19 remained fractional in the presence of the Fab, while the rate of Hub19 loss was slowed only at higher concentrations. The RO and rate of the monomer loss for the Fab did not change in the presence of Hub19 at 37 °C at low concentrations, whereas at 50 °C, the RO decreased from 2.7 ± 0.8 to 1.25 ± 0.1, along with a slight increase in the rate. Thus, the impact on Fab by Hub19 was likely through a similar mechanism as for Hub07 at 50 °C, with promotion of unimolecular unfolding, while the unimolecular unfolding of Hub19 was also slowed at the highest concentration. However, at 37 °C, Hub19 and Fab did not appear to affect each other very much at lower concentrations.

For 37 °C at higher concentrations, the rate of the Fab monomer loss increased significantly in the presence of Hub19. Most of this difference related to the slowing of the Fab monomer loss at high concentrations in the single-protein formulations, suggesting a self-protective effect due to macromolecular crowding or similar mechanism as reported previously.^30^ Conversely, the Hub19 monomer loss was slowed in the coformulations and remained essentially unimolecular. Thus, the higher protein concentrations also appeared to produce a similarly protective effect on the unfolding of Hub19.

## Discussion

Protein medicines of different modality can potentially be premixed into a coformulated product that then requires a higher level of analysis for quality control.^7,9^ The overall stability and the change in stability for each component must be carefully investigated to ensure the safety and efficacy of the coformulated drug molecules. Assuming that the overall stability of a coformulation is governed by the least stable component, four possible scenarios exist even with a simple two-protein coformulation, whereby the overall stability is (1) lower than both isolated protein components; (2) the same as the least stable component when measured in isolation; (3) in between the stabilities of the two isolated components; and (4) higher than both isolated components. In these scenarios, the shelf-life of the coformulated product would be as follows: shorter than the shelf-life of the least stable protein component (scenario 1); the same as that of the least stable component (scenario 2); in between the shelf-life of the two components (scenario 3); and longer than that of the most stable component (scenario 4). To determine how each of the two proteins change their stability within the formulations for each scenario requires a careful analysis of the stability of each component before and after mixing.

In this study, the degradations of two specific coformulation systems, Hub07–Fab and Hub19–Fab, were characterized under two stressed conditions of 37 and 50 °C, respectively. Hub07 and Fab did not show significant unfolding at these temperatures from their thermal unfolding curves (Figure S2). Hub19 indicated the potential for a minor fraction of unfolding at 50 °C. Thus, we observed the degradation of the single-protein and coformulation systems due to interactions under near-native-structure conditions. From our observations of the rate orders for the monomer loss, we proposed that Fab, Hub07, and Hub19 can each form partially unfolded species (PUS: Fab*, Hub07*, and Hub19* in Figure 6) in a rate-limiting step toward aggregation. These PUS could initiate the aggregation cascades through homomolecular interactions and potentially also via heteromolecular interactions.

Previously, the Fab in this study was used to stabilize a therapeutic IgG1 molecule against the monomer loss with the same Fab sequence.^30^ Hub07 and Hub19 have more construct complexity and less sequence similarity than the previously studied IgG1, bringing additional uncertainties for the Fab to coformulate and stabilize. Moreover, Hub07 and Hub19 antibodies were more aggregation-prone than Fab. The behaviors of Hub07–Fab and Hub19–Fab coformulations both fell into Scenario 3 whereby the overall stability was greater than that for the least stable components when measured in isolation (Hub07 or Hub19) alone but less stable than the most stable component (Fab). This could manifest through interactions between the two protein components, or simply by crowding through an overall increase in protein concentration. Crowding has the potential to stabilize some proteins through suppressing partial unfolding but could also destabilize some by promoting the higher-order interactions that lead to aggregation.

The Fab has the following properties from earlier investigations that may make it an ideal coformulation partner: (1) thermally stable with a *T*_m_ of over 80 °C; (2) self-stabilizing at higher concentrations; and (3) formation of reversible small aggregates that prevent further unfolding and aggregation.^31^ There are two proposed mechanisms through which Fab could stabilize Hub07 and Hub19: (1) direct interaction with Hub07 and Hub19 to mask or suppress the solvent exposure of any aggregation-prone patches and (2) crowding to suppress the Hub07/Hub19 partial unfolding events that lead to aggregate formation.

The stabilizations shown in Hub07–Fab and Hub19–Fab coformulations were different. The rate of Hub07 degradation in the presence of the Fab was reduced but followed a similar exponential kinetic profile and an unchanged reaction rate order. This indicated that Fab could reduce the frequency of effective collisions for Hub07 in solution to slow down aggregation without a change in the mechanism. We propose that Fab could achieve this by crowding effects that slowed the rate of partial unfolding of Hub07 to Hub07*.

By comparison, the Hub19–Fab coformulations showed a more complex behavior, with exponential kinetics in isolation, and also for the coformulation at 37 °C, but with evidence of a lag phase in aggregation kinetics in the coformulated Hub19 at 50 °C. At 37 °C, Fab stabilized Hub19 against the monomer loss as their overall concentrations increased, but the rate of the monomer loss for the Fab increased along with the RO for the Fab at high concentrations. This indicated that the interaction between Hub19 and Fab was more than simply a crowding effect. The rate of the monomer loss for Hub19 appeared to be slowed by the formation and presence of higher-order Fab species at higher concentrations, potentially protecting Hub19 through increased crowding from the larger species or through a direct interaction.

At 50 °C, the Fab inhibited the Hub19 monomer loss up to day 30, but this lag phase was then followed by a rapid monomer loss of Hub19. The overall kinetics were slightly slower on average in the coformulation, and the reaction rate order on Hub19 at 50 °C was not changed. Meanwhile, the rate order on the Fab decreased from 2.7 to 1.25 at 50 °C, mainly due to an increase in the rate of the Fab monomer loss at lower concentrations. This suggests that an interaction between Fab and Hub19 at lower concentrations promoted the Fab monomer loss but suppressed the Hub19 monomer loss. However, after an initial lag period, this led to a critical nucleus formation and more rapid Hub19 monomer loss.

One might conclude that Hub19 followed distinct kinetic pathways for aggregation at 37 and 50 °C such that the effect from the addition of the Fab was different. Thermal unfolding data showed that Hub19 had a *T*_m_ less than 70 °C in PBS (Figure S2). As the stress temperature of 50 °C was closer to the Hub19 *T*_m_ than for any other species, a small amount of partial unfolding may have promoted the formation of the aggregate. The Fab at 50 °C would then have a greater potential to interact with the Hub19 PUS species than at 37 °C.

Scenario 4 in which both proteins are stabilized by their intermolecular interactions would be the ideal as the proteins could be simply mixed at their clinically approved concentrations without extra concern for their overall stability. However, in practice, as demonstrated here, the situation can be more complex and should be analyzed in a case-specific manner.

## Conclusions

Only a few previous studies have looked into the stability of the coformulation of antibodies.^6,8,9,32^ This specific Fab interacted with Hub07 or Hub19 to increase their stability, as we observed previously for a more closely related IgG1.^30^ Acting as an aggregation decelerator, the use of a bioactive Fab represents a promising coformulation component for antibody candidates.

## Supporting Information Available

SEC analyses for day 0 and day 40/60; thermal unfolding profiles for single proteins and coformulations; static light-scattering thermograms for single proteins and coformulations; and reaction-order plots for single-protein and coformulation experiments (PDF)
