# Supplementary material for: Stability Convergence in Antibody Coformulations
Source: Mol Pharm. 2022 Oct 20;19(11):4098–110. doi: 10.1021/acs.molpharmaceut.2c00534 (PMC9644375; doi:10.1021/acs.molpharmaceut.2c00534)
Supplement: Supplementary file 1 — mp2c00534_si_001.pdf [file mp2c00534_si_001.pdf]

Supporting information

Stability convergence in antibody coformulations

Hongyu Zhang[1,2], Paul A. Dalby[1,*]

1 Department of Biochemical Engineering, UCL, London, UK, WC1E 6BT

2 EPSRC Future Targeted Healthcare Manufacturing Hub, UCL, London, UK, WC1E 6BT

*Correspondence to: p.dalby@ucl.ac.uk

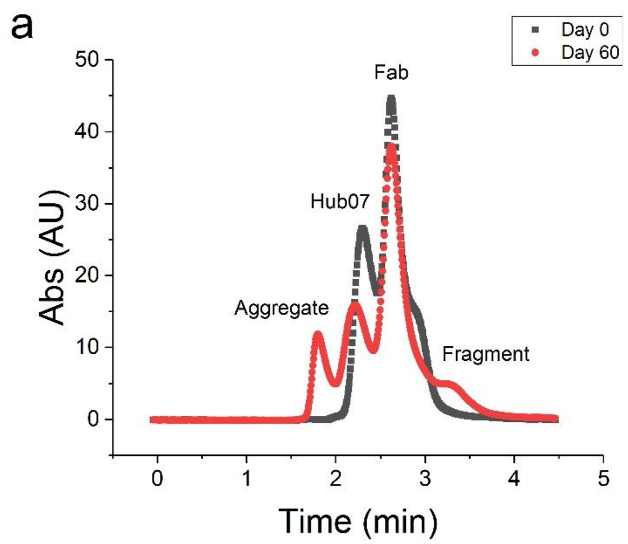
a
Day 0
Day 60
Fab
Hub07
Aggregate
Fragment
Abs (AU)
Time (min)
50
40
30
20
10
0
0
1
2
3
4
5

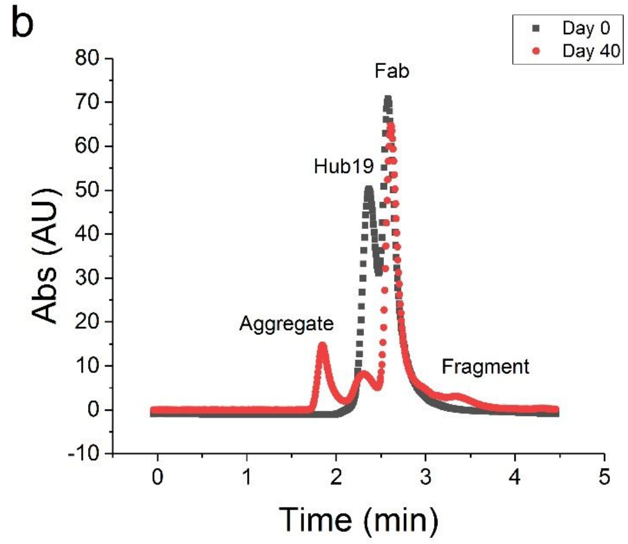
b
Day 0
Day 40
Fab
Hub19
Aggregate
Fragment
Abs (AU)
Time (min)
80
70
60
50
40
30
20
10
0
-10
0
1
2
3
4
5

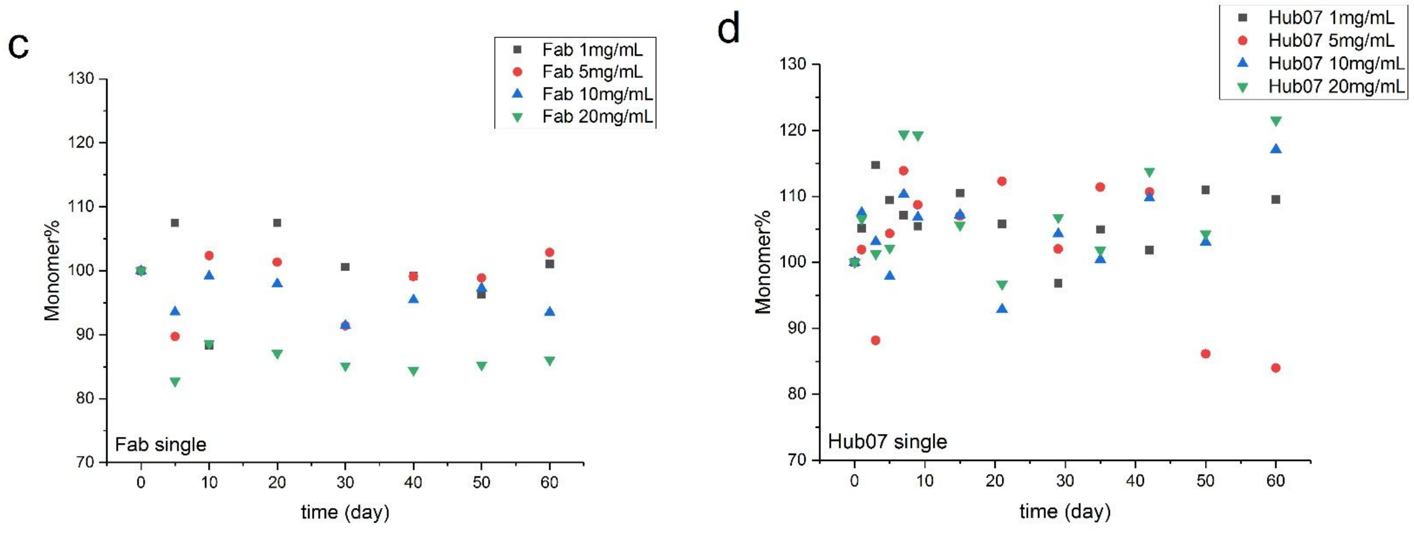
c
Fab 1mg/mL
Fab 5mg/mL
Fab 10mg/mL
Fab 20mg/mL
Monomer%
Fab single
time (day)
130
120
110
100
90
80
70
0
10
20
30
40
50
60
d
Hub07 1mg/mL
Hub07 5mg/mL
Hub07 10mg/mL
Hub07 20mg/mL
Monomer%
Hub07 single
time (day)
130
120
110
100
90
80
70
0
10
20
30
40
50
60

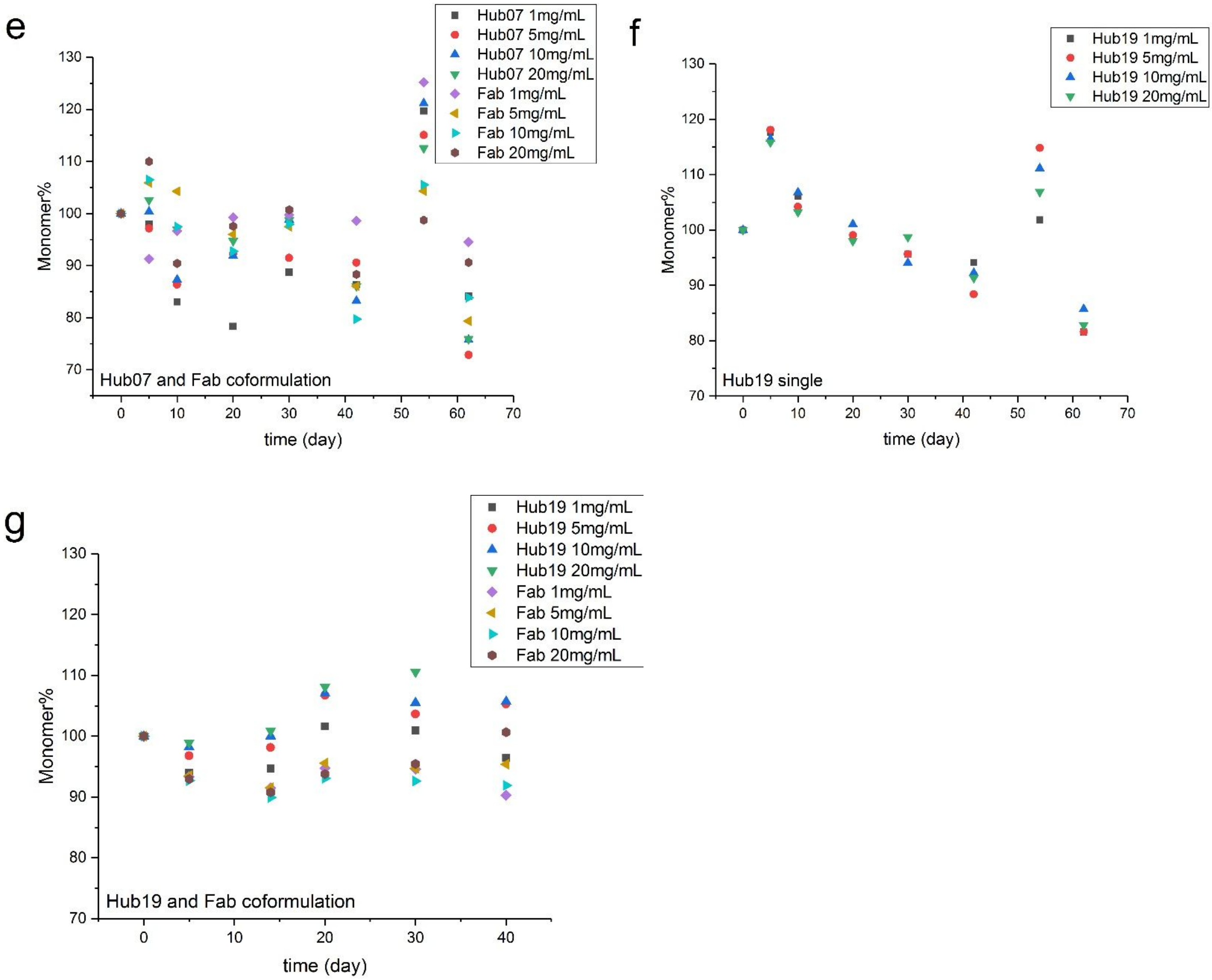

Figure S1: SEC of Hub07-Fab (a) and Hub19-Fab (b) coformulations in PBS buffer (pH7.4) for day 0 and day 40/60. Here the 1 mg/mL data are shown as example. Aggregate species is the major degradation product compared to a minor fragment species. Minor peak position change in Hub07 before and after stressing could be a physical effect through transient interactions either with the column matrix or in the original sample. The monomer changes at 4 °C for single and coformulation experiments are shown in (c-g) as a reference.

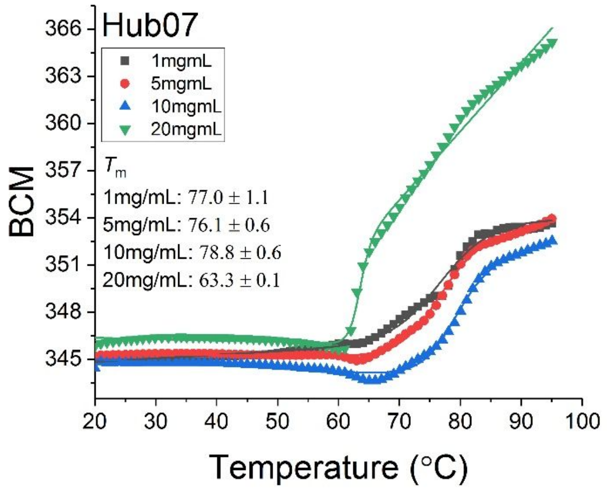
Hub07
1mgmL
5mgmL
10mgmL
20mgmL
T_m
1mg/mL: 77.0 ± 1.1
5mg/mL: 76.1 ± 0.6
10mg/mL: 78.8 ± 0.6
20mg/mL: 63.3 ± 0.1
BCM
Temperature (°C)

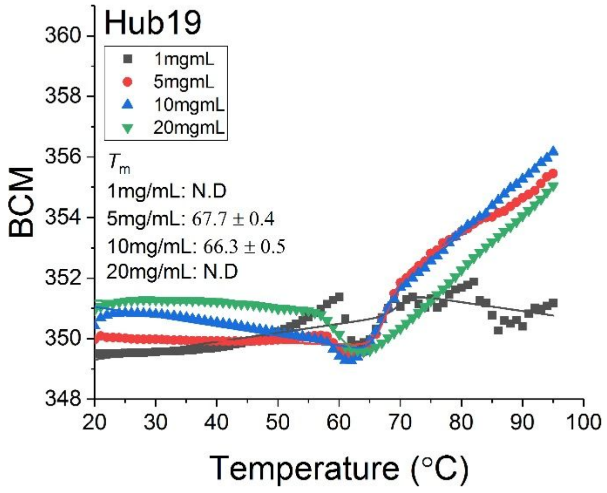
Hub19
1mgmL
5mgmL
10mgmL
20mgmL
T_m
1mg/mL: N.D
5mg/mL: 67.7 ± 0.4
10mg/mL: 66.3 ± 0.5
20mg/mL: N.D
BCM
Temperature (°C)

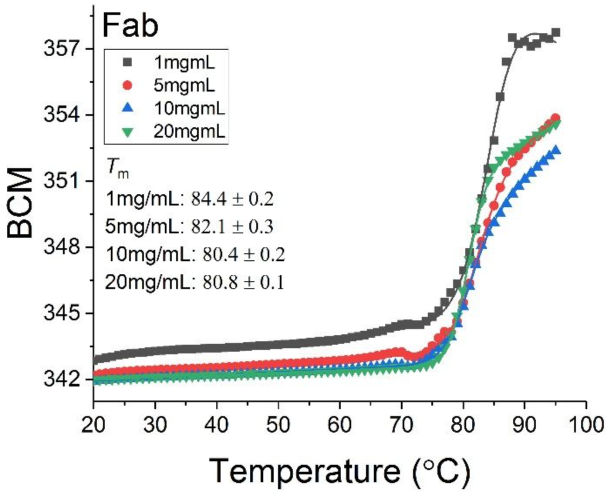
Fab
1mgmL
5mgmL
10mgmL
20mgmL
T_m
1mg/mL: 84.4 ± 0.2
5mg/mL: 82.1 ± 0.3
10mg/mL: 80.4 ± 0.2
20mg/mL: 80.8 ± 0.1
BCM
Temperature (°C)

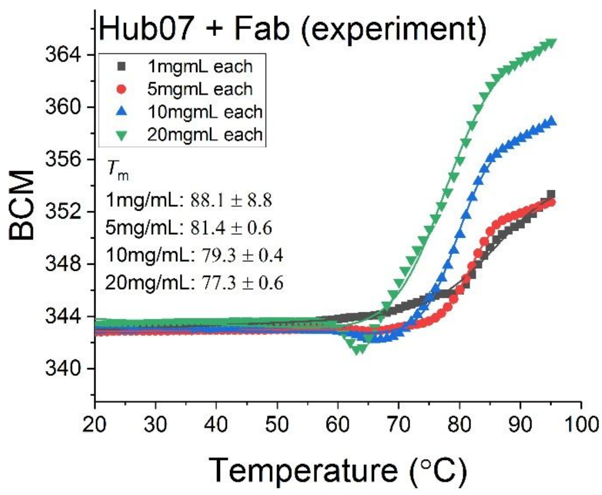
Hub07 + Fab (experiment)
1mgmL each
5mgmL each
10mgmL each
20mgmL each
T_m
1mg/mL: 88.1 ± 8.8
5mg/mL: 81.4 ± 0.6
10mg/mL: 79.3 ± 0.4
20mg/mL: 77.3 ± 0.6
BCM
Temperature (°C)

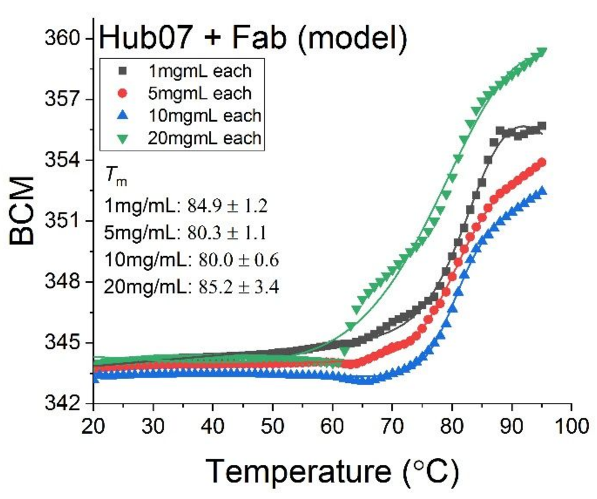
Hub07 + Fab (model)
1mgmL each
5mgmL each
10mgmL each
20mgmL each
T_m
1mg/mL: 84.9 ± 1.2
5mg/mL: 80.3 ± 1.1
10mg/mL: 80.0 ± 0.6
20mg/mL: 85.2 ± 3.4
BCM
Temperature (°C)

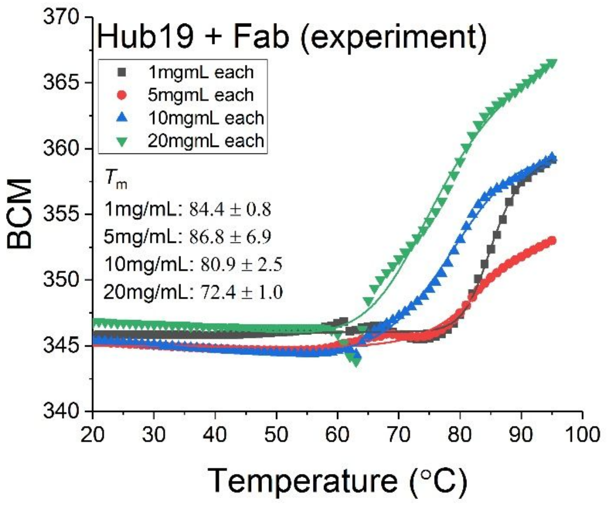
Hub19 + Fab (experiment)
1mgmL each
5mgmL each
10mgmL each
20mgmL each
T_m
1mg/mL: 84.4 ± 0.8
5mg/mL: 86.8 ± 6.9
10mg/mL: 80.9 ± 2.5
20mg/mL: 72.4 ± 1.0
BCM
Temperature (°C)

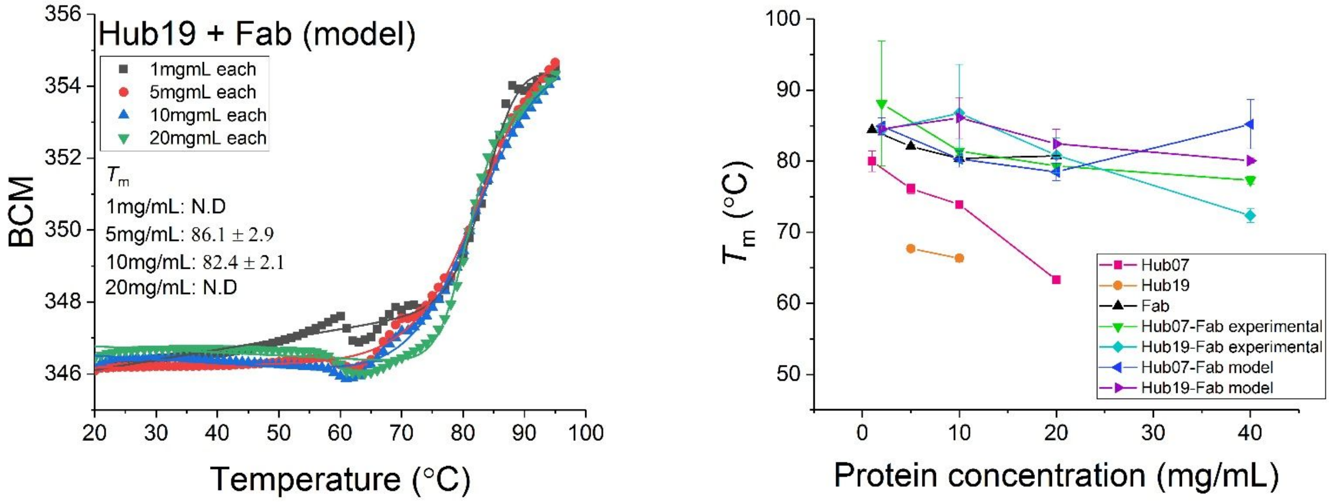

Figure S2: Typical thermal unfolding profiles for single proteins and coformulations of Hub07, Hub19 and Fab in PBS, pH 7.4. The change in fluorescence against rise in temperature is reported as BCM (Barycentric Mean, the centre of the mass of fluorescence emission). The continuous lines represent the best fit of the data to a two-state unfolding model (while some data appeared to be three-state, the fitting could not converge due to insufficient signal on the minor transition). Each experiment was repeated in triplicate. Coformulation experiment data are shown in comparison with the modelled data, which results from the mathematical average of Hub07/19 and Fab single-protein data. Error bars shown are standard deviations from triplicate fits.

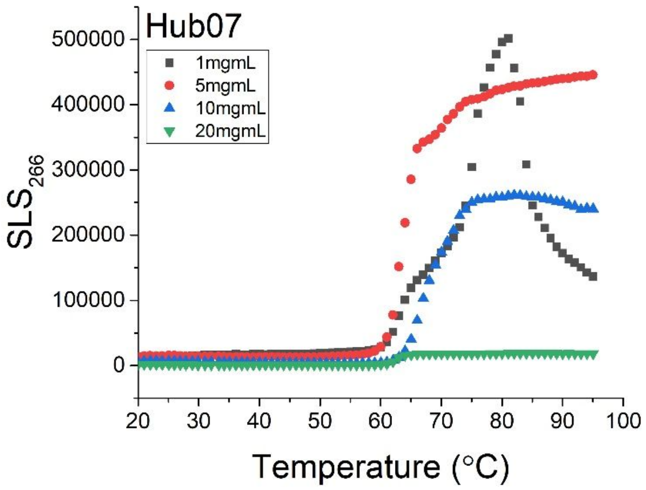

Hub07
1mgmL
5mgmL
10mgmL
20mgmL
SLS266
Temperature (°C)

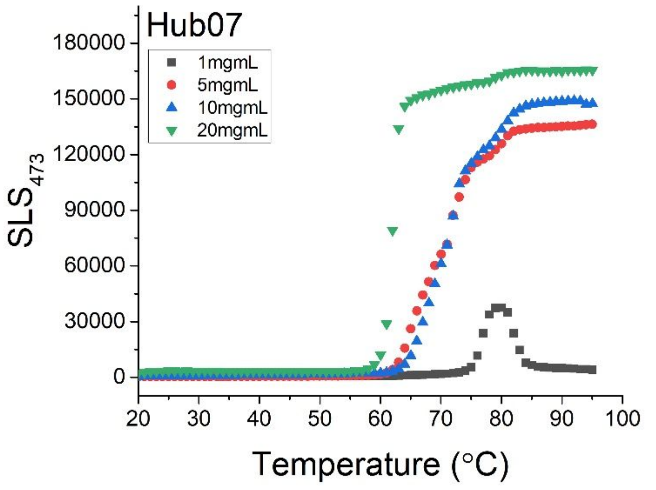

Hub07
1mgmL
5mgmL
10mgmL
20mgmL
SLS473
Temperature (°C)

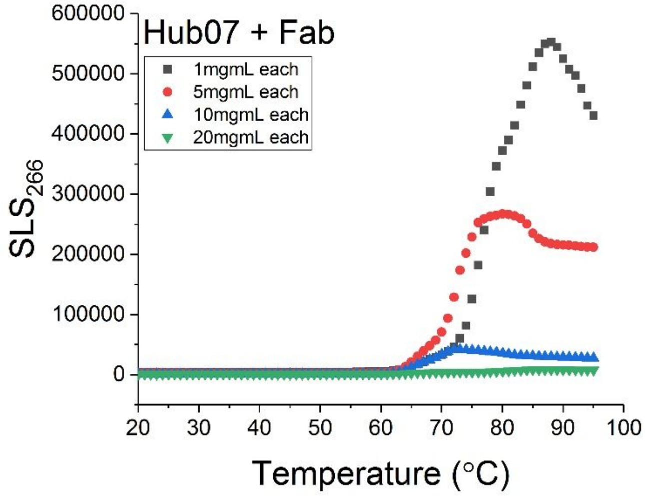

Hub07 + Fab
1mgmL each
5mgmL each
10mgmL each
20mgmL each
SLS266
Temperature (°C)

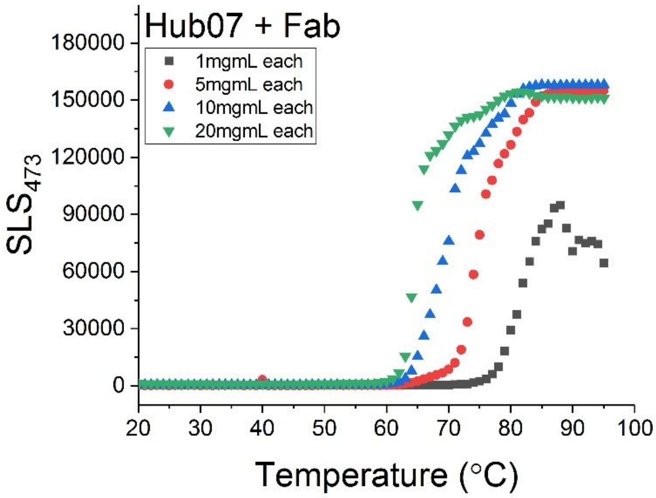

Hub07 + Fab
1mgmL each
5mgmL each
10mgmL each
20mgmL each
SLS473
Temperature (°C)

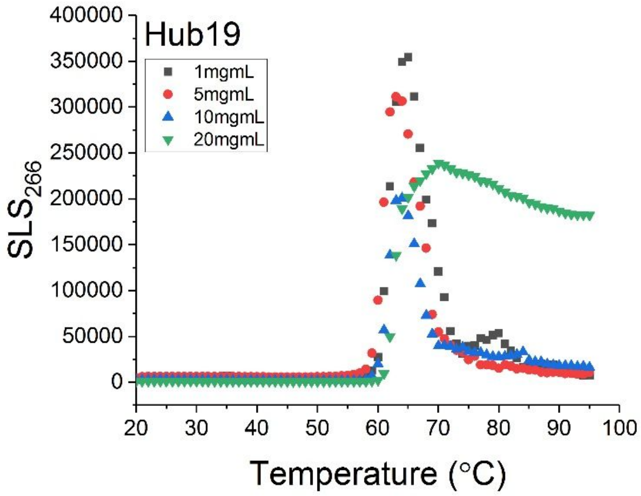

Hub19
1mgmL
5mgmL
10mgmL
20mgmL
SLS266
Temperature (°C)

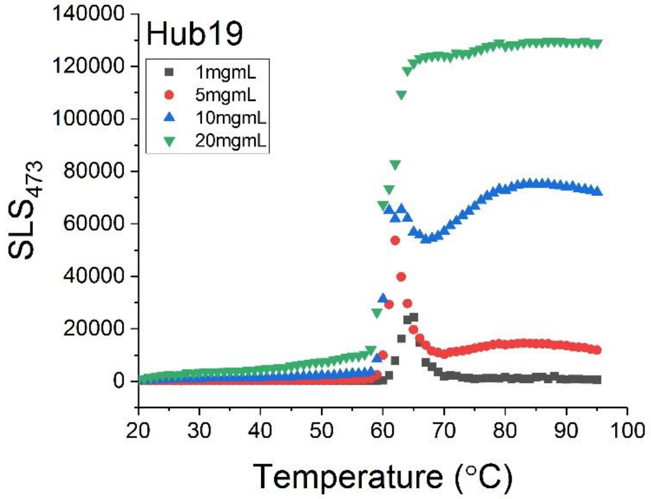

Hub19
1mgmL
5mgmL
10mgmL
20mgmL
SLS473
Temperature (°C)

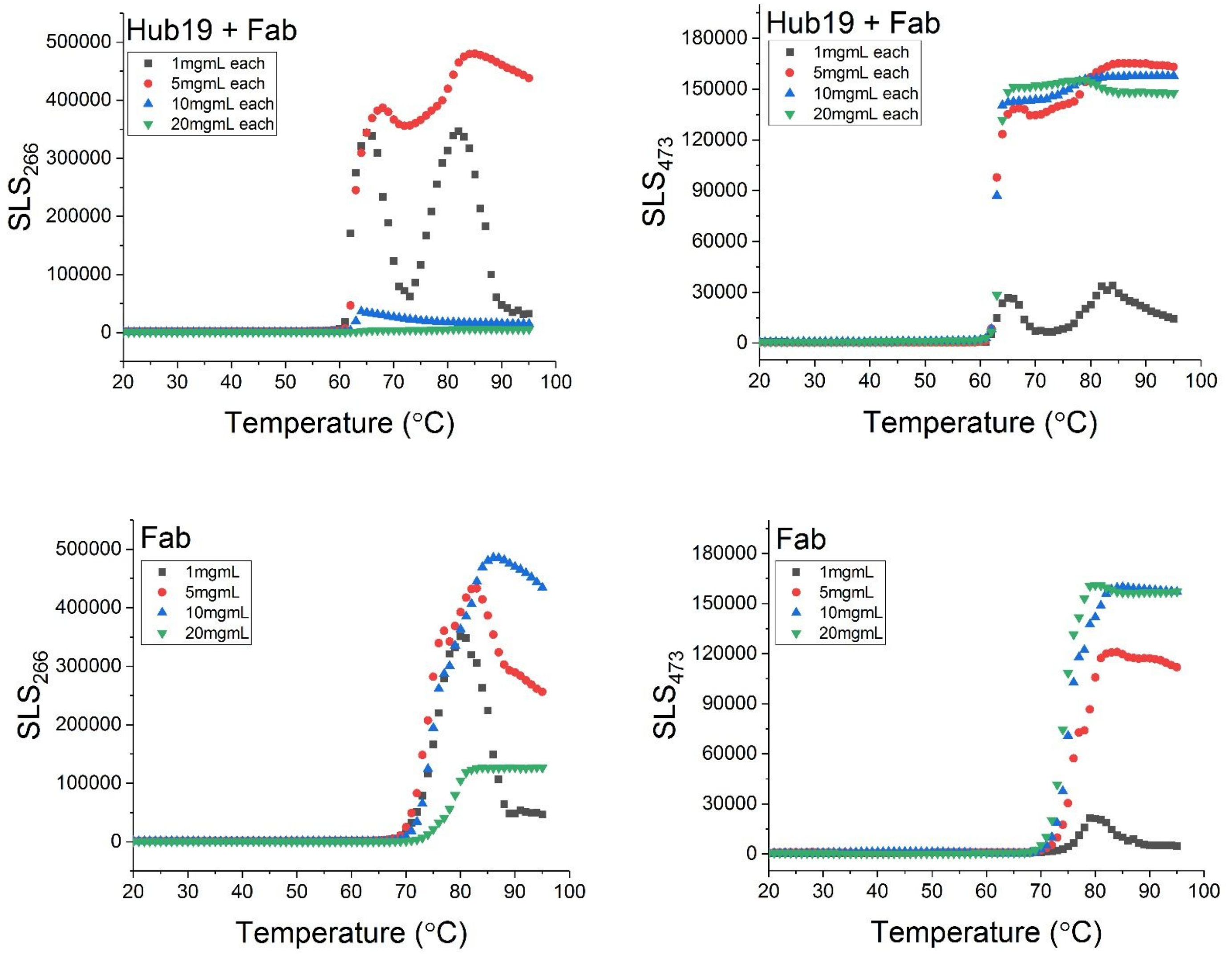

Figure S3: Typical static light-scattering (SLS) thermograms for single proteins and coformulations. Increases in smaller and larger aggregate particles are reported by SLS at 266 nm and 473 nm, respectively.

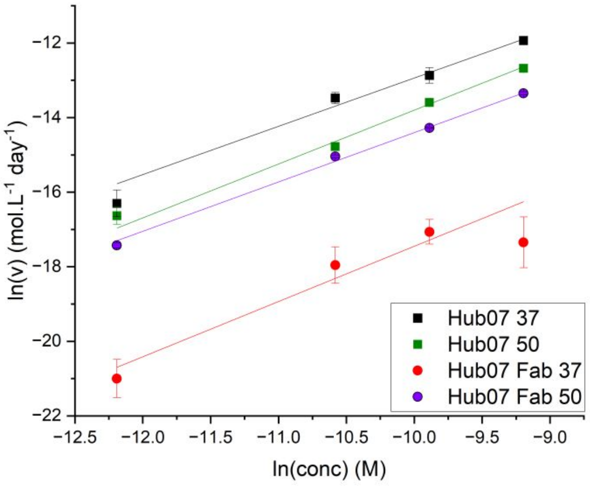

A

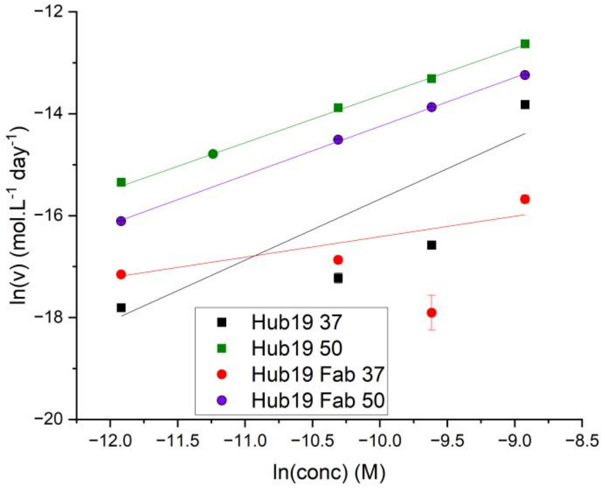

B

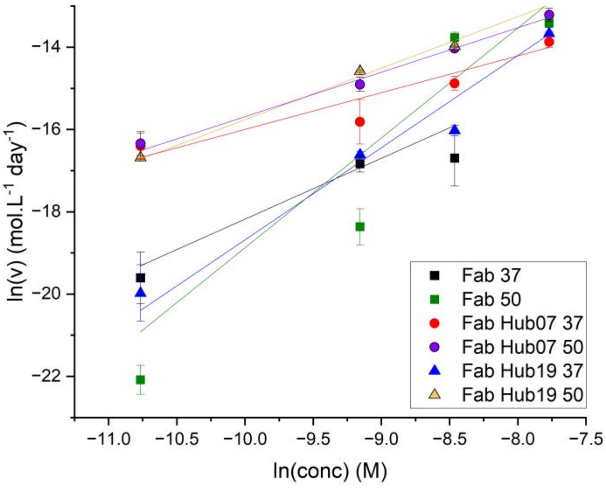

C

Figure S4: Change in reaction order for A) Hub07, B) Hub19 and C) Fab in single and coformulation experiments. The solid lines represent weighted linear fits to the data. Data of Fab including 20 mg/mL were fitted separately (not shown, slope = 0.3 ± 0.9).
